# The Additive Value of Radiomics Features Extracted from Baseline MR Images to the Barcelona Clinic Liver Cancer (BCLC) Staging System in Predicting Transplant-Free Survival in Patients with Hepatocellular Carcinoma: A Single-Center Retrospective Analysis

**DOI:** 10.3390/diagnostics13030552

**Published:** 2023-02-02

**Authors:** Mohammad Mirza-Aghazadeh-Attari, Bharath Ambale Venkatesh, Mounes Aliyari Ghasabeh, Alireza Mohseni, Seyedeh Panid Madani, Ali Borhani, Haneyeh Shahbazian, Golnoosh Ansari, Ihab R. Kamel

**Affiliations:** Russell H. Morgan Department of Radiology and Radiological Sciences, Johns Hopkins University School of Medicine, Baltimore, MD 21205, USA

**Keywords:** hepatocellular carcinoma, MRI, BCLC, cancer, radiomics, transplant

## Abstract

Background: To study the additive value of radiomics features to the BCLC staging system in clustering HCC patients. Methods: A total of 266 patients with HCC were included in this retrospective study. All patients had undergone baseline MR imaging, and 95 radiomics features were extracted from 3D segmentations representative of lesions on the venous phase and apparent diffusion coefficient maps. A random forest algorithm was utilized to extract the most relevant features to transplant-free survival. The selected features were used alongside BCLC staging to construct Kaplan–Meier curves. Results: Out of 95 extracted features, the three most relevant features were incorporated into random forest classifiers. The Integrated Brier score of the prediction error curve was 0.135, 0.072, and 0.048 for the BCLC, radiomics, and combined models, respectively. The mean area under the receiver operating curve (ROC curve) over time for the three models was 81.1%, 77.3%, and 56.2% for the combined radiomics and BCLC models, respectively. Conclusions: Radiomics features outperformed the BCLC staging system in determining prognosis in HCC patients. The addition of a radiomics classifier increased the classification capability of the BCLC model. Texture analysis features could be considered as possible biomarkers in predicting transplant-free survival in HCC patients.

## 1. Introduction

Hepatocellular carcinoma is a major contributor to the cancer-associated global disease burden, with an estimated incidence of more than one million cases annually [1]. It is predicted that a shift will occur in the epidemiologic pattern of HCC, with chronic conditions playing a more important role than infectious agents [2,3,4]. This transition will necessitate a thorough overhaul of existing diagnostic and therapeutic practices associated with the care of HCC [5]. Furthermore, HCC is one of the most heterogeneous malignancies of the gastrointestinal tract, both in regard to the genetic makeup of each lesion and the baseline clinical characteristics of the patients involved [6,7]. Thus, it is necessary to consider a wide spectrum of data pertaining to a lesion when designing diagnostic, therapeutic, and prognostic guidelines.

Diagnostic imaging is used for initial evaluation and risk stratification of incidental lesions and has also been considered in criteria dictating therapeutic measures for HCC patients [8]. Liver transplantation in carefully selected patients has been considered the therapeutic measure with the highest long-term survival and lowest recurrence rate in HCC [9]. However, based on the Milan criteria, it is reserved for subjects with single tumor lesions with a diameter measuring less than 5 cm or 2–3 tumors, each measuring less than 3 cm without vascular invasion [10]. Other guidelines have also been suggested, all of which incorporate tumor volume and other semantic imaging biomarkers. The Barcelona Clinic Liver Cancer (BCLC) Staging System is one of such criteria which encompasses imaging findings and important biologic-functional correlates such as the Child–Pugh classification and the Eastern Cooperative Oncology Group score (ECOG score) [11]. Although the BCLC Staging System has proven to be one of the most utilized staging systems, it is faced with significant limitations [12]. The two most notable are the possibility of offering curative treatment exclusively to early-stage lesions [13,14], and the inherent tumoral heterogeneity (due to tumor burden, liver function status, the underlying cause of cancer, and different molecular and cellular pathways of oncogenesis) witnessed in stage B and C patients which is not represented via any metrics in this classification system [11].

Recent progress in quantitative imaging has ushered in a new discipline of incorporating a more diverse and comprehensive set of biomarkers into staging algorithms and decision support systems [15], which can more appropriately represent the immense heterogeneity seen in the histological, pathological, and clinical profile of different tumoral lesions [15,16]. Multiple studies have previously reported the net benefit of adding semantic imaging signs such as tumor margin (expressed as a dichotomous variable) or volumetric measures into BCLC classification [17,18].

Agnostic imaging features, otherwise known as radiomics features, are mathematically generated, high-dimension, quantitative descriptors which are extracted from medical images and are utilized to obviate the necessity of considering important texture parameters, which may act as phenotypic proxy indicators of complex pathologic processes and underlying genomic variations [19]. Importantly, a rigorously validated radiomics protocol has the potential to be a proxy indicator of genetic anomalies, functioning as a means of virtual tissue sampling. Currently, the additive value of radiomics features to clinical risk stratification criteria is rarely explored, and to the best of our knowledge, there are no such initiatives regarding risk stratification of hepatocellular carcinoma.

The present article aims to study the additive value of radiomics features to the BCLC staging system in clustering HCC patients based on transplant-free survival.

## 2. Materials and Methods

### 2.1. Ethical Considerations

The present study was approved by the institutional review board of the medical center where it was performed, and the need for informed consent was waived as the survey was being done as a retrospective analysis of anonymized patient medical records. This study complied with the Health Insurance Portability and Accountability Act.

### 2.2. Study Population

The present retrospective, single-center study was conducted on patients with a diagnosis of hepatocellular carcinoma (HCC) presenting to our tertiary cancer referral center between 2006 and 2018. Inclusion criteria consisted of patients having a histopathologically-proven HCC who underwent baseline MR imaging prior to initiation of therapy. Exclusion criteria consisted of patients with incomplete hepatic MRI protocols, active lesions, or metastasis from an active malignancy other than HCC, and patients with incomplete clinical profiles. The sample size was determined based on an analysis performed in a previous study, where the minimal sample size was determined to be 242 patients [17].

### 2.3. Clinical Medical Records of Patients

Electronic medical records containing data pertaining to the most recent and all previous visits up to 5 years were accessed, and demographic, laboratory, and clinical data were extracted [20]. Relevant data for HCC cancer staging was also extracted [20], including functional status, the BCLC staging, and Child–Pugh [21].

### 2.4. Imaging Study

A standardized imaging protocol was applied to all patients based on institutional guidelines:

A 1.5-T MR scanner (Avanto, Siemens Healthcare) with a phased-array torso coil was utilized to obtain detailed liver/lesion images. The routine institutional protocol consisted of T2-weighted turbo spin-echo images (matrix size, 256 × 256; slice thickness, 8 mm; interslice gap, 2 mm; repetition time [TR]/echo time [TE], 4500/92 msec; receiver bandwidth, 32 kHz) and breath-hold DWI echo-planar images with spectral fat saturation technique (matrix size, 128 × 128; slice thickness, 8 mm; interslice gap, 2 mm; b value of 50 and 750 s/mm^2^; TR, 3000 msec; TE, 69 msec; receiver bandwidth, 64 kHz). Breath-hold unenhanced and contrast-enhanced (0.1 mmol/kg intravenous gadopentetate dimeglumine; Magnevist; Bayer) T1-weighted 3-dimensional fat-suppressed spoiled gradient-echo images (field of view, 320–400 mm; matrix size, 192 × 160; slice thickness, 2.5 mm; TR, 5.77 msec; TE, 2.77 msec; receiver bandwidth, 64 kHz; flip angle, 10) in the hepatic arterial (20 s), portal-venous (70 s), and delayed (3–5 min) phase were also obtained. Acquisition trajectory of the k-space was set to cartesian, and the number of excitations was 3–4.

ADC values were determined using the least-square exponential fitting of all b values within the predetermined range of b values. The equation used to calculate the ADC value is as the following: ADC = −ln (S/S0)/b, where S0 is the signal intensity of no diffusion gradients and b is the b value [22].

Read More: https://www.ajronline.org/doi/10.2214/AJR.13.11818 (accessed on 19 January 2023)

### 2.5. Imaging Data Analysis

Lesion segmentation was performed by a post-doctoral research fellow with 5 years of experience under the oversight of an expert board-certified radiologist with 25 years of experience. The MR Multiparametric prototype (version 3, Siemens Healthcare, Malvern, PA, USA) was used for the volumetric analysis of the ADC maps. All lesions were segmented on 2D planes, and respective 3D models composed of a series of these 2D label maps were used to extract agnostic features. The portal-venous phase image, the DWI with the lowest acquired b value (b = 50 s/mm^2^), and the ADC map were loaded into the software. DWI images were elastically coregistered to the respective portal-venous phase images, and the resulting transformation was also applied to the ADC map [23,24]. Segmentation was performed manually on the portal-venous phase, and then the contours were transferred to other sequences, applying manual correction when necessary.

The ADC maps and DW images were selected for feature selection because of the fact that previous studies had shown their possible beneficence in representing information about the tumor microenvironment [25]. The portal-venous phase images were selected as this sequence is important in the initial diagnosis, management, and follow-up of hepatocellular carcinoma, as a change in the enhancement of the tumor in this phase is shown to be associated with clinical outcome and response to therapy [26,27]. The dominant lesion was segmented in each of the patients, and features were extracted from the respective contour. When multiple lesions were confluent, an arbitrary boundary around the dominant lesion (the lesion with the biggest diameter) was used to extract features. Segmentations were saved in the NRRD (“nearly raw raster data”) format, and the contours were used for feature extraction without any further interpolation.

### 2.6. Feature Extraction

Radiomics features were extracted from manually designated 3D contours. An in-house developed program based on MATLAB (2022a, Mathworks Inc., Natick, MA, USA), combined with the texture analysis toolbox (https://github.com/mvallieres/radiomics, accessed on 9 August 2022) was utilized to extract radiomics features. This toolbox extracted 95 radiomics features from venous-enhanced images and ADC maps. Features belonged to either first, second, or third-order radiomics features. More in this regard is presented in Tables S1 and S2 of the Supplementary Files. The codes used to generate the features were IBSI compliant [28].

### 2.7. Feature Selection and Supervised Learning Protocol

A random forest (RF) classification algorithm was used for the determination of the best set of radiomics features in determining prognosis in HCC patients. Fine-tuning of the RF protocol was performed to enable the model to detect the most pertinent features. Based on the RF classifier’s structure, features situated close to the model’s root have superior utility compared to those located at the leaf nodes, meaning there is an inverse relationship between the depth of the feature and its overall importance in the task of classification. The RF model was developed using the following specifications: number of trees = 3000, node size = 6, mtry = 60. Out-of-bagging estimates measure (OOB) is utilized to determine the prediction error of the model. OOB uses the sample data and performs subsampling, creating training datasets for the model to learn from. More information regarding the structure of the models is presented in the supplementary file.

The random forest model was developed using the open-source R statistical software, with the following packages being used in different stages of model development and testing included: “randomForestSRC,” “tidyverse,” “naniar”, “ggRandomForests”, “prodlim”, “survival”, “riskRegression”, and “survminer”.

### 2.8. Statistical Analysis

SPSS software (Version 23.0.0, IBM, Boston, MA, USA) was used for the analysis of demographic and clinical parameters. The normality of variables was assessed by the Kolmogorov–Smirnov test, and appropriate statistical tests were used for univariate associations. Student t-tests and chi-square tests were used for continuous and categorical parameters, respectively. Kaplan–Meier curves were utilized to estimate survival.

All *p* values were considered statistically significant at *p* < 0.05.

## 3. Results

**Figure 1:**
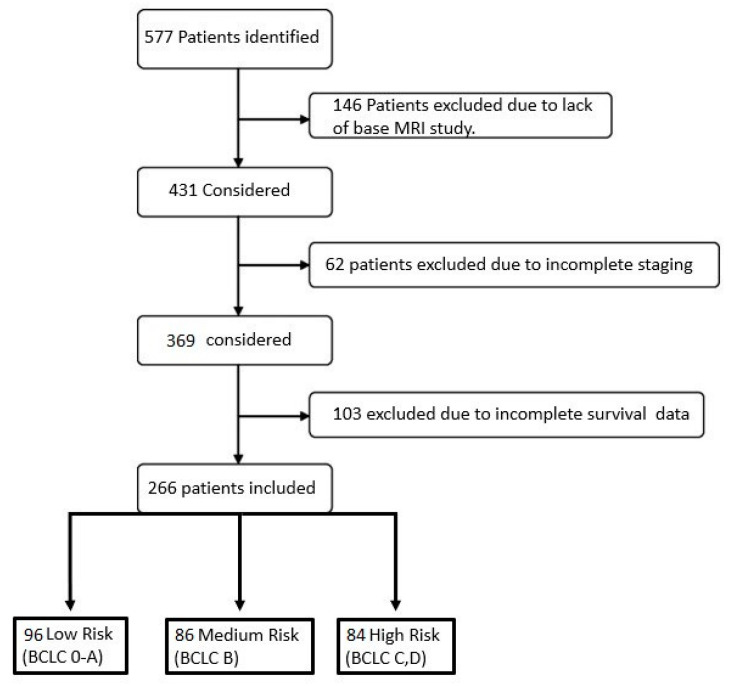
Flow chart of the patients included in the present study.

A total of 266 patients were included in the final analysis. Figure 1 demonstrates the flow chart of the study. Demographic information of patients is presented in Table 1. Patients were classified into three clusters based on their respective BCLC stage: Stage 0–1 was defined as the low-risk group, Stage 2 patients were defined as medium risk, and patients with Stage 3–4 were defined as the high-risk group. These three groups were not significantly different regarding age, gender, and the prevalence of cirrhosis. The performance status of patients in the first group was significantly lower than those in the high and medium-risk groups (*p* = 0.001). Portal invasion was significantly higher in the high-risk group compared to the low-risk and medium-risk groups (*p* = 0.001). AFP was also significantly higher in this group. However, there were no significant differences in serum albumin, bilirubin, and INR.

**Figure 2:**
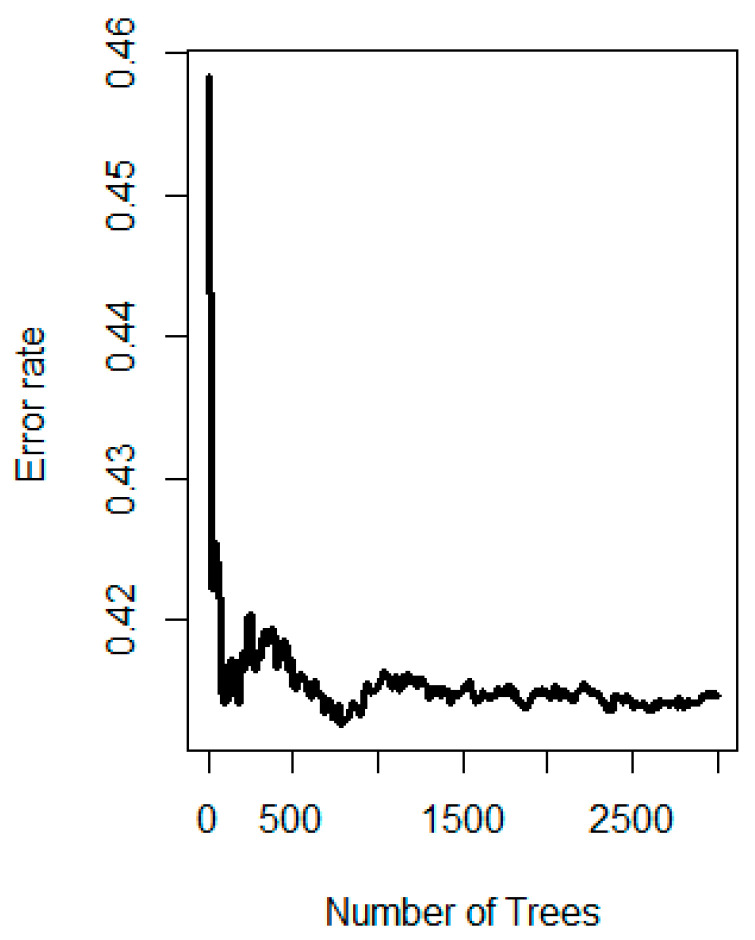
Error rate plotted against number of trees used in the random forest protocol.

**Figure 3:**
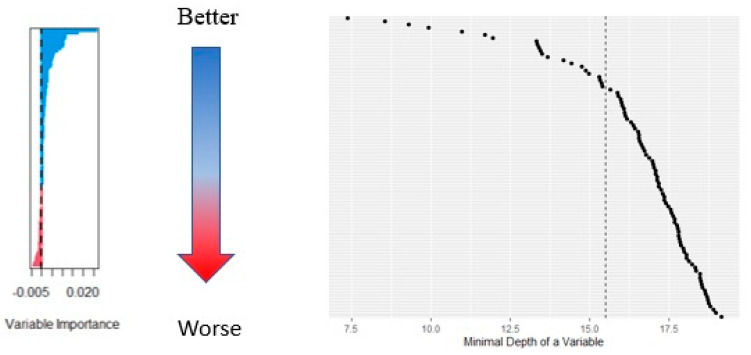
Relative importance of the radiomics features included in the study (**Left**), and the minimal depth of the variables in the random forest model (**right**).

**Figure 4:**
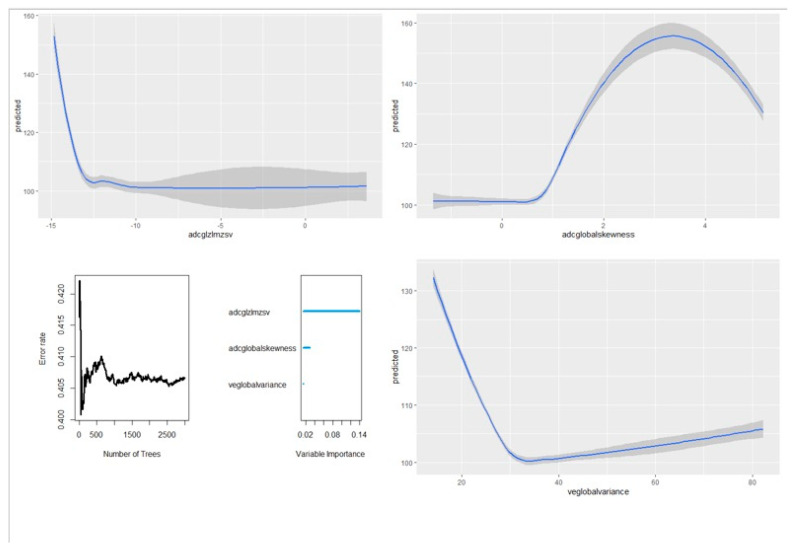
Partial variable dependence plots of the 3 selected radiomics.

Figure 2 also demonstrates the effect of adding trees on the error rate of the classification. Figure 3 presents the set of variables, their respective depth, and their importance. Nested RFs built by adding variables based on the descending order of importance showed that just the top three most important features, consisting of veglobalvariance, adcglzlmzsv, and adcglobalskewness, showed great performance (see Supplementary Figure S1). A complete list of features and their relative importance is included in Table S2 of the Supplementary File. Partial variable dependence plots of the three selected features are presented in Figure 4.

**Figure 5:**
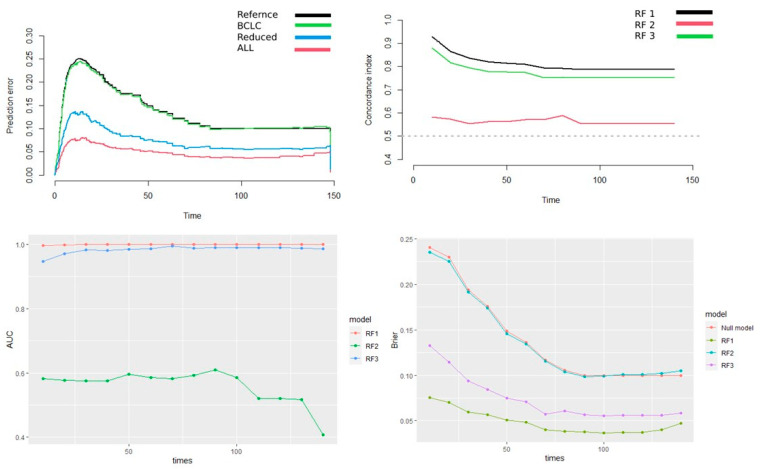
Top, left: Prediction error of the 3 models included in the analysis (ALL: Combined model, BCLC: model only incorporating BCLC, reduced: Model only including radiomics features). Top right: Concordance index of the 3 models (RF1: Combined model, RF2: BCLC, RF3: model using only radiomics). Bottom, left: Area under the curve during time of the 3 models (RF1: Combined model, RF2: BCLC, RF3: model using only radiomics). Bottom, right: Brier score of the models over time (RF1: Combined model, RF2: BCLC, RF: model using only radiomics).

We utilized RF to develop three clustering models, a model constructed using the three most important radiomics features, a model based on BCLC alone, and a combined model which incorporated both previously mentioned. Figure 5 depicts the perdition error curves of the three models. The combined model achieved the lowest Integrated Brier score (0.048), followed by the radiomics-only model (0.072) and the BCLC-only model (0.135). Lower scores are associated with higher accuracy. Figure 5 also depicts the concordance index of the three models, and as witnessed, the radiomics model closely resembles the combined model and significantly outperforms the BCLC model. This Figure also depicts the AUC of the model plotted over time, and the calibration curves of the models, showing the superiority of the radiomics and combined models compared to the BCLC model.

**Figure 6:**
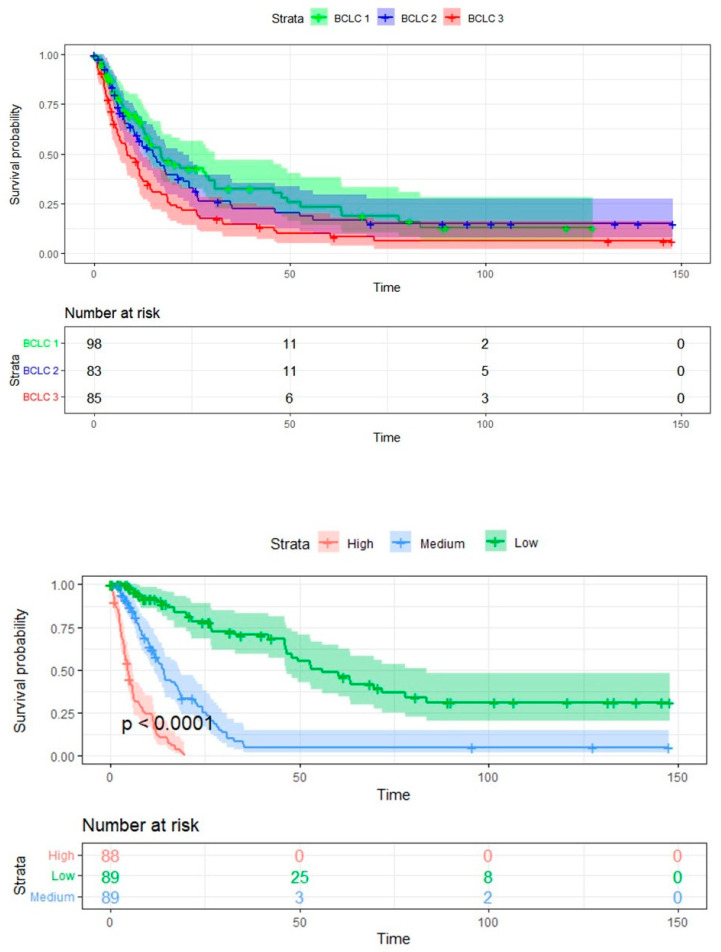
Above: Kaplan–Meier curve of the combined model, incorporating radiomics features and BCLC classification (BCLC 1 = 0-A, BCLC 2 = B, BCLC 3 = C-D) with its respective *p* value. Below: Kaplan–Meier curve of the radiomics classifier based on three categorical tertiles with its respective *p* value (low, medium, and high radiomics score).

Figure 6 shows the Kaplan–Meier curve of the combined model, which includes patient classification based on BCLC and Radiomics score. This Figure also depicts the Kaplan–Meier curve when the rad score is used as a three-level categorical value. As seen in the Figure, there was a statistically significant difference between the survival of patients when they were classified into three groups based on radiomics score, which was more significant compared to the difference seen with BCLC staging.

## 4. Discussion

In the present article, we show the benefit of carefully adding selected texture analysis radiomics features into one of the most widely used classification schemes, BCLC. We show that not only do three carefully selected features—veglobalvariance, adcglzlmzsv, and adcglobalskewness—enhance the BCLC criteria, but they can outperform it even when utilized separately to develop a classifier. Previous studies have shown the utility of first-order features such as variance and skewness and second-order features such as grey-level zone length matrix in predicting the clinical profile of patients with HCC, such as aiding in classifying patients based on local control and survival [24]. It is hypothesized that certain genetic alterations are responsible for poor clinical outcomes, such as those related to uncontrolled tumoral growth and distant metastasis; however, these alterations can cause phenotypic changes visible on anatomic levels, which can be readily detected by texture features, thus enabling them to act as proxy indicators of genetic alterations [29].

The BCLC lexicon is highly regarded as one of the most comprehensive tools in risk stratification and prognostication of patients with HCC [11]. This lexicon utilizes clinical, functional, and semantic imaging features of HCC patients in a stepwise manner to suggest optimal treatment strategies and also provides estimations on survival [11,30]. However, the intrinsic variability in each of these input data may limit the extent to which the criteria perform precisely and accurately. Regarding clinical input, the BCLC model has been shown to underperform in clinical scenarios where patients have multiple lesions and undergo locoregional therapy for a single lesion. In these instances, progression may have numerous patterns with significantly different late clinical outcomes [31,32]. Furthermore, the BCLC criteria do not consider the variability seen in the clinical profile of a patient when a new lesion emerges. Overall the case-by-case heterogeneity seen in progression patterns in relation to patient profile warrants further inclusion of more informative data into decision-making models [33,34]. On the other hand, relying on basic quantitative imaging metrics such as size or objective parameters such as semantic features of the lesion included in BCLC may also be subject to the same insufficiencies [12].

Such significant limitations include the fact that tumor diameter in lesions under 5 cm does not seem to be as strongly correlated to survival as thought before [35,36], and also the emergence of new therapeutic modalities which rely on quantifying the extent of extrahepatic disease, which necessitates a revisit and reevaluation of BCLC criteria C, as patients with extrahepatic involvement are categorically selected for systemic therapy with chemotherapeutic agents rather than locoregional therapy [36].

Ghasabeh et al. have shown the utility of incorporating more reproducible metrics, such as baseline ADC values of lesions and tumor margin, into BCLC and the Cancer of the Liver Italian Program (CLIP) [17]. The inclusion of these two imaging features was able to increase the predictive function of BCLC and CLIP up to 9% and 6%, respectively.

Radiomics features [19] could be a suitable supplement to the BCLC criteria, as these features have been shown to act as proxy indicators of genetic heterogeneity in cancers, which to a large extent, is the culprit for the immense heterogeneity seen in morphology and natural history of cancer [37,38]. On the other hand, experiments with radiomics features have shown the critical importance of combining radiomics features with the clinical data of patients in generating optimal classifiers [39]. Furthermore, having a rigorously validated radiomics tool may be utilized as a virtual biopsy, obviating the need for taking multiple tissue samples for tasks related to the clinical management of patients [40].

Fang et al. conducted a study where clinical data of patients were combined with radiomics-generated scores to predict progression-free survival in patients with HCC. They created a nomogram based on BCLC, AFP levels, and radiomics signature, which was able to outperform a model generated only on AFP and BCLC. This was evident by the higher C scores of the combined model vs. the clinical model (0.821 (95% CI: 0.726–0.915) vs. 0.76 (95% CI: 0.667–0.851)). Similar results were also reported by Liu et al. They, too, combined BCLC, albumin, and bilirubin values with a radiomics score generated via LASSO selection method and then utilized it to classify patients into dichotomous categories based on progression-free survival. The standalone radiomics score was able to achieve a sensitivity of 0.946 and specificity of 0.544 in classifying patients into poor outcome and good outcome groups. The overall accuracy of a radiomics model was superior to the clinical model, though without a statistically significant relation (0.638 vs. 0.681). A combined model based on radiomics score and clinical data significantly outperformed both standalone models (sensitivity of 0.784, specificity of 0.737, accuracy of 0.745). Kong et al. reached similar conclusions regarding the prognostic implications of a combined clinical model in regard to response to TACE as well. However, these studies had certain limitations that limited the generalizability of their results. In all of them, the sample size in the validation set was small, ranging between 30 and 50 cases. Furthermore, in the nomograms constructed by all the previous groups, BCLC was given a relatively small significance in stratification, and in all of the cases, the BCLC classification was reduced to a dichotomous outcome (Group B vs. Group C), not incorporating the various nuances seen when the original classification if applied.

Another interesting observation is reported by Li et al., who utilized radiomics features extracted from [18F] FDG PET/CT images of BCLC 0 and A stages to develop a nomogram to classify patients based on disease-free survival. They performed univariate cox regression analysis on multiple clinical parameters to determine the best variables to include in their nomogram and reported that conventional staging schemes such as BCLC and Child–Pugh were not of statistical significance to include in their model, while a radiomics score constructed on the aforementioned extracted features was able to increase the predictive capabilities of a model only trained on clinical variables, such as INR, the existence of microvascular invasion and serum total bilirubin [41]. This article highlights two important findings: the reproducibility of radiomics features in multimodality imaging and its consistent performance in early-stage HCC cases. Importantly, radiomics features were used as a reliable marker when there was minimal tumor burden, highlighting the possibility of using radiomics features in the early stages of HCC.

Our study provides substantial evidence regarding the possible benefit of incorporating radiomics features into clinical risk-stratifying schemes such as BCLC. We show that a carefully selected set of radiomics features are able to outperform the conventional risk stratification modalities by a significant margin. Furthermore, to the best of our knowledge, our efforts are one of the only few which have focused on combining clinical HCC cancer classification systems, such as BCLC, with radiomics features, a methodologic consideration that generates added value to both clinical and radiomics staging schemes.

However, our conclusions should be viewed in light of the limitations of our study. First of all, we included patients from a single tertiary cancer referral center. Thus, our results may not be generalizable to front-line management of HCC, especially in cases where limited imaging and clinical data are extracted from patients. Notably, due to the limited number of patients in our institution, we were not able to externally validate our model. We also included the single largest lesion from each patient, which would result in information pertaining to other satellite lesions being lost [42].

Furthermore, we only extracted a limited number of radiomics features from each 3D contour applied to two imaging sequences and then utilized a machine learning protocol to develop a clustering model. The utilization of radiomics features extracted from deep algorithms may provide a more representative marker for the development of classifiers. Importantly, we extracted features from a segmentation that was determined by a junior and senior-level radiologist concurrently; thus, the impact of inter-reader contouring variability on RF reproducibility was not assessed. Features selected based on our methodologies may be subject to variation when different readers draw different contours [43,44]. It is also worthy of notice that issues regarding pre-processing of images, such as registration and histogram normalization, could contribute to reduced reproducibility [45]. Alleviating these challenges may also enable the utilization of a comprehensive set of imaging sequences to extract a more representative set of features.

In the present study, we only included the added benefit of radiomics to BCLC and no other risk-stratifying tools such as CLIP, which intrinsically contain more information regarding tumor burden and may be possible candidates for integration with radiomics features. Including other risk stratification methods, combined with radiomics features extracted from other imaging sequences such as T2WI, or even other imaging modalities (for example, combined texture analysis of CT, MR, and PET images) may further improve the prognostic potential of combined texture-clinical models [46].

Furthermore, it is essential to mention that in the present article, we utilized transplant-free survival as a means to determine the predictive efficacy of our model, and other measures of survival were not included in our analysis.

## 5. Conclusions

A combined radiomics-BCLC model can cluster HCC patients based on transplant-free survival with superior functionality compared to BCLC alone. The same radiomics signature can also cluster patients into three groups with a significant difference in transplant-free survival. Radiomics features have the potential to be used as additive biomarkers to already established risk stratification schemes.

## Figures and Tables

**Table 1:** Demographic, biochemical lab results, clinical staging, and semantic imaging findings of patients included in the present study.

			Low Risk (BCLC 0,1)	Medium Risk (BCLC 2)	High Risk (BCLC 3,4)	*p* Value
**Number (%)**			96 (36.0%)	86 (32.3%)	84 (31.5%)	N/A
**Gender (Male, Female)**			71(74%), 25(26%)	69(80.2%), 17 (19.8%)	70 (83.3%), 14(16.7%)	0.290
**Age (Mean ± SD)**			66.6 ± 10.8	64.6 ±10.5	64.1± 9.8	0.677
**Cirrhosis (%)**			77 (80.2%)	61 (70.9%)	64 (76.2%)	0.343
**Ascites (%)**			26 (27.1%)	35(40.7%)	38 (45.3%)	0.04
**Transplant (Yes, No)**			51 (53.1%), 45 (46.9%)	15 (17.4%), 71 (82.6%)	7 (8.3%), 77 (91.7%)	0.0001
**Event (Transplant or Death)**			54 (56.3%)	60 (68.9%)	72 (85.7%)	N/A
**Performance Status**		0	68 (70.0%)	42 (48.8%)	28 (33.3%)	0.001
		1	27 (29.0%)	37 (43.0%)	42 (50.0%)	
		2	1 (1.0%)	7 (8.2)	10 (11.9%)	
		3	0 (0.0%)	0 (0.0%)	4 (4.7%)	
**Underlying Hepatic Condition**		ALD	10 (10.4%)	15 (17.4%)	12 (14.3%)	0.390
		Hepatitis C	57 (59.4%)	37 (43%)	36 (42.9%)	0.036
		Hepatitis B	12(12.5%)	6 (7.0%)	15 (17.9%)	0.09
		Other	2 (2.1%)	7 (8.1%)	5 (6.0%)	*N/A*
**Child’s Classification**	Class A		73 (76%)	54 (62.8%)	51 (60.7%)	*N/A*
	Class B		23 (24%)	32 (37.2%)	29 (34.5%)	
	Class C		0	0	4 (4.%)	
**Okuda Classification**	Stage I		59 (61.2%)	34 (39.5%)	39 (46.4%)	*N/A*
	Stage II		35 (36.5%)	38 (44.2%)	32 (38.1%)	
	Stage III		2 (2.1%)	14 (16.3%)	13 (15.5%)	
**CLIP Score**	0		48 (50%)	0	4 (4.8%)	N/A
	1		37 (38.5%)	29 (33.7%)	9 (10.7%)	
	2		9 (9.4%)	33 (38.4%)	30 (35.7%)	
	3		2 (2.1%)	20 (23.3%)	25 (29.8%)	
	4		0	4 (4.7%)	12 (14.3%)	
	5		0	0	3 (3.6%)	
	6		0	0	1 (1.2%)	
**Tumor Margin**	Well defined		67 (69.8%)	53 (61.6%)	63 (75%)	0.001
	Ill-defined		29 (30.2%)	33 (38.4%)	21 (25%)	
**Portal Invasion**			8(8.3%)	14 (16.3%)	70 (83.3%)	0.001
**Varices**			4 (4.1%)	7 (8%)	11 (13.0%)	0.09
**AFP (Mean ± SD)**			1544.4 ± 4776	5676 ± 16654	13179 ± 39141	0.002
**Albumin**			3.6 ± 0.7	3.59 ± 0.73	3.56 ± 0.67	0.3
**Bilirubin**			1.29 ± 1.27	1.78 ± 2.1	1.70 ± 2.1	0.4
**INR**			1.15 ± 0.29	1.2 ± 0.23	1.15 ± 0.19	0.7

## Data Availability

All data will be made available based on request from the corresponding author. Code used for radiomics features extraction is available in the link provided in the supplementary file.

## Supplementary Materials

The following supporting information can be downloaded at: https://www.mdpi.com/article/10.3390/diagnostics13030552/s1, Figure S1. The relation between number of variables included in the random forest model and the prediction error rate. As seen in the image, the optimal point was achieved with 3 variables, and inclusion of further variables did not significantly contribute to the model; Figure S2: Kaplan–Meier curve demonstrates the transplant-free survival of patients based on BCLC clustering alone; Table S1: Radiomics features extracted, and their relative importance in the random forest classification [47]; Table S2: Classification of radiomics features extracted in the present article.
