# Supplementary material for: The Additive Value of Radiomics Features Extracted from Baseline MR Images to the Barcelona Clinic Liver Cancer (BCLC) Staging System in Predicting Transplant-Free Survival in Patients with Hepatocellular Carcinoma: A Single-Center Retrospective Analysis
Source: Diagnostics (Basel). 2023 Feb 2;13(3):552. doi: 10.3390/diagnostics13030552 (PMC9914401; doi:10.3390/diagnostics13030552)
Supplement: Supplementary file 1 [file diagnostics-13-00552-s001.zip › Supplementaryhcc .pdf]

Supplementary material

2. Methods and Materials:

2.1. Grey level Discretization before extraction of radiomics features: To determine if radiomics features were dependent on the number of gray levels (Ng), radiomics features were extracted with resampled Ng values of 16, 32, 64, 128, and 256. Of these, 64 was the optimal value in our analysis and provided the best performance, and was used all of the respective analysis. A bin width of 25 HU was used to extract the features and symmetrical GLCM was enforced. The intensity range of the images were between 0-1200. More information regarding the exact parameters used in feature extraction could be fined in the link bellow (an open source package for Matlab (1): https://www.mathworks.com/matlabcentral/fileexchange/51948-radiomics?s_tid=FX_rc1_behav). All code and data are available using GitHub directories. (https://github.com/mvallieres/radiomics)

2.2. The radiomics quality of the present article was 9 (25%). The complete report is attached as a separate PDF file.

Table S1. Radiomics features extracted, and their relative importance in the random forest classification.

Feature Name	Importance	Relative importance	Feature Name	Importance	Relative importance
adcglzlmzsv	0.0264	1.0000	veglrlmrlv	0.0026	0.0990
adcglrlmlrhge	0.0171	0.6475	adcglzlmlzlge	0.0023	0.0856
veglobalvariance	0.0115	0.4362	adcglrlmsrhge	0.0022	0.0848
adcglobalskewness	0.0114	0.4301	adcglrlmlgre	0.0020	0.0744
veglrlmhgre	0.0105	0.3964	adcglrlmgln	0.0018	0.0699
veglzlmlgze	0.0104	0.3949	veglzlmlze	0.0018	0.0664
veglzlmlzlge	0.0101	0.3820	veglzlmlzhge	0.0016	0.0619
tumorprinicpalaxislength3	0.0089	0.3367	bclccodecombine3group	0.0015	0.0550
adcglzlmgln	0.0075	0.2825	veglrlmlrlge	0.0013	0.0501
adcglzlmhgze	0.0057	0.2173	vengtdmcontrast	0.0013	0.0477
adcglzlmzsv	0.0264	1.0000	vengtdmstrength	0.0012	0.0472
adcglrlmlrhge	0.0171	0.6475	veglzlmglv	0.0012	0.0461
veglobalvariance	0.0115	0.4362	veglrlmlrhge	0.0011	0.0436
veglzlmszlge	0.0056	0.2119	veglrlmsre	0.0011	0.0404
veglobalskewness	0.0049	0.1846	adcngtdmbusyness	0.0008	0.0317
veglrlmlgre	0.0047	0.1768	adcglrlmrln	0.0008	0.0290
veglrlmsrlge	0.0044	0.1684	adcglrlmsre	0.0008	0.0286
adcglzlmglv	0.0042	0.1590	adcglzlmlzhge	0.0007	0.0268
veglzlmzsv	0.0032	0.1218	adcngtdmcoarseness	0.0007	0.0247
adcglrlmhgre	0.0028	0.1055	veglcmautocorrelation	0.0006	0.0243
veglcmenergy	0.0027	0.1041	tumorsurfacearea	0.0003	0.0107
veglrlmglv	0.0027	0.1020	veglrlmgln	0.0002	0.0094
tumorprinicpalaxislength1	0.0027	0.1007	veglobalkurtosis	0.0002	0.0093
adcglzlmszhge	0.0006	0.0218	adcngtdmstrength	0.0002	0.0092
veglcmcontrast	0.0005	0.0208	veglrlmlre	0.0002	0.0090
vengtdmcomplexity	0.0005	0.0191	tumorvolume	0.0002	0.0086
adcglrlmrp	0.0004	0.0160	veglrlmrln	0.0002	0.0075
adcglrlmlrlge	0.0004	0.0150	veglzlmhgze	0.0001	0.0047
adcglzlmlze	0.0004	0.0141	tumorprinicpalaxislength2	0.0001	0.0040
veglzlmzp	0.0004	0.0137	adcglcmdissimilarity	0.0001	0.0034
adcglrlmsrlge	0.0003	0.0130	tumorsurfacearea	0.0003	0.0107
adcglrlmrlv	0.0003	0.0129	adcglcmenergy	-0.0003	-0.0096
veglcmentropy	0.0003	0.0125	veglcmhomogeneity	-0.0003	-0.0096
veglrlmrp	0.0001	0.0032	adcglrlmlre	-0.0004	-0.0142
veglrlmsrhge	0.0000	0.0012	vengtdmbusyness	-0.0004	-0.0156
adcglcmhomogeneity	0.0000	-0.0005	adcngtdmcontrast	-0.0005	-0.0190
veglcmcorrelation	-0.0001	-0.0024	adcglcmautocorrelation	-0.0006	-0.0215

veglcmsumaverage	-0.0001	-0.0025	adcglcmcorrelation	-0.0006	-0.0221
veglcmdissimilarity	-0.0001	-0.0031	adcglcmcontrast	-0.0008	-0.0305
adcglrlmglv	-0.0001	-0.0037	adcglcmentropy	-0.0008	-0.0308
vengtdmcoarseness	-0.0002	-0.0069	veglzlmzsn	-0.0008	-0.0314
adcglzlmzp	-0.0010	-0.0373	veglzlmsze	-0.0009	-0.0325
adcglcmvariance	-0.0011	-0.0401	veglzlmszhge	-0.0009	-0.0335
adcglzlmlgze	-0.0011	-0.0415	veglzlmgln	-0.0014	-0.0547
adcglzlmsze	-0.0014	-0.0524	adcglcmsumaverage	-0.0015	-0.0550
adcngtdmcomplexity	-0.0014	-0.0531	adcglzlmzsn	-0.0022	-0.0826
adcglzlmzp	-0.0010	-0.0373	adcglobalvariance	-0.0024	-0.0912
veglcmvariance	-0.0031	-0.1161	adcglzlmszlge	-0.0027	-0.1036
adcglobalkurtosis	-0.0035	-0.1335	tumorsolidity	-0.0043	-0.1619

Table S2. Classification of radiomics features extracted in the present article.

**Feature class**	**Feature Name**
**Gray Level Run Length Matrix Features (GLRLM)**	Short-run emphasis (SRE), Long-run Emphasis (LRE), Gray-level non-uniformity (GLN), Run-length non-uniformity (RLN), Run Percentage (RP), Low Gray-level Run Emphasis (LGRE), High Gray-level Run Emphasis (HGRE), Short Run Low Gray-level Emphasis (SRLGE), Short Run High Gray-level Emphasis (SRHGE), Long Run Low Gray-level Emphasis (LRLGE), Long Run High Gray-level Emphasis (LRHGE), Gray-level Variance (GLV), and Run Length Variance (RLV)
**Gray Level Size Zone Matrix Features (GLSZM)**	Small Zone Emphasis (SZE), Large Zone Emphasis (LZE), Gray-level non-uniformity (GLN), Zone Size non-uniformity (ZSN), Zone percentage (ZP), Low Gray-level Zone Emphasis (LGZE), High Gray-level Zone Emphasis (HGZE), Small Zone Low Gray-level Emphasis (SZHGE), Large Zone Low Gray-level Emphasis (LZLGE), Large Zone High Gray-level Emphasis (LZHGE), Gray-level Variance (GLV), and Zone Size Variance (RLV)
**Gray Level Co-occurrence Matrix Features (GLCM)**	Contrast, Correlation, Energy, Variance, Sum average, Dissimilarity, Autocorrelation, Entropy, and Homogeneity
**Global Features**	Mean, Maximum, and Minimum (for both ADC and VE), Tumor Solidity, Surface Area, and Volume
**Histogram-based Features**	Variance, Skewness, and Kurtosis
**Neighborhood Gray-tone Difference Matrix (NGTDM)**	Mean, Variance, Kurtosis, Strength, and Skewness

Figure S1. The relation between number of variables included in the random forest model and the prediction error rate. As seen in the image the optimal point was achieved with 3 variables and inclusion of further variables did not significantly contribute to the model.

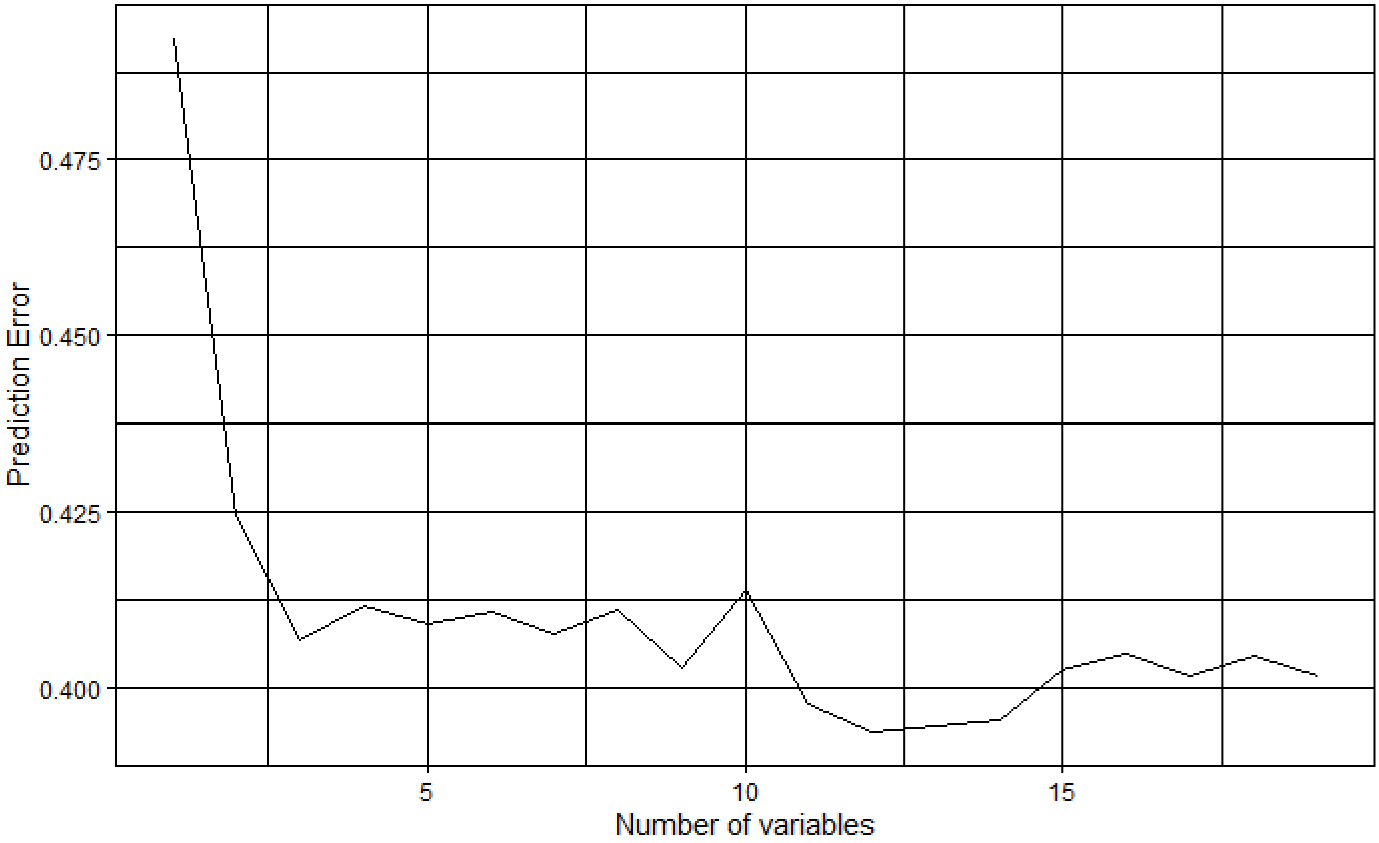

Figure S2. Kaplan Meier curve demonstrates the transplant-free survival of patients based on BCLC clustering alone.

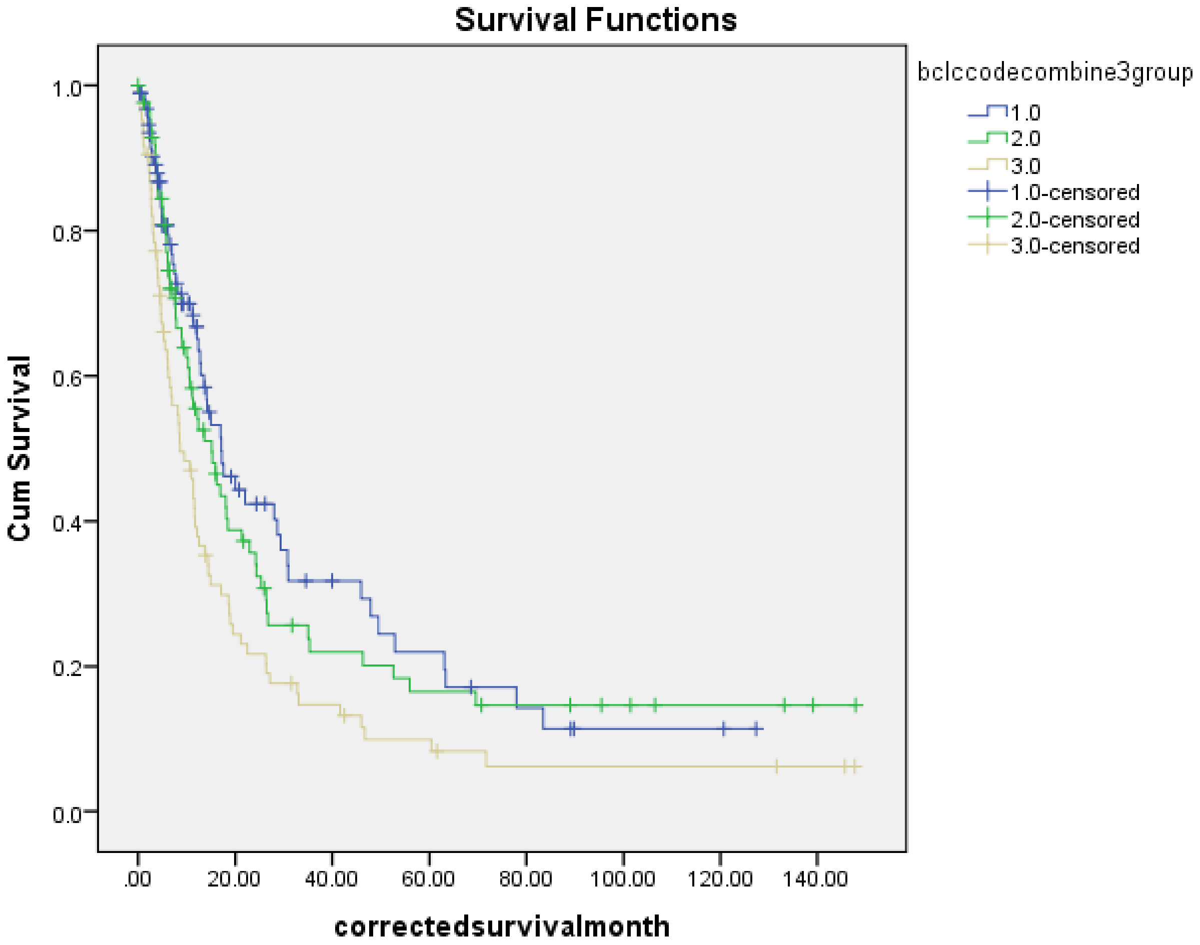

References:

1. Vallières M, Freeman CR, Skamene SR, El Naqa IJPiM, Biology. A radiomics model from joint FDG-PET and MRI texture features for the prediction of lung metastases in soft-tissue sarcomas of the extremities. 2015;60(14):5471.
